# Facile Recycling of Waste Biomass for Preparation of Hierarchical Porous Carbon with High-Performance Electromagnetic Wave Absorption

**DOI:** 10.3390/molecules29112455

**Published:** 2024-05-23

**Authors:** Yihui Zhou, Jingjing He, Jiafu Hong, Haihe Xie, Xuexia Lin

**Affiliations:** 1 College of Chemical Engineering, Huaqiao University, 668 Jimei Avenue, Xiamen 361021, China; xh19990429@163.com (Y.Z.); hjfstudy@163.com (J.H.); 2 School of Mechanical, Electrical & Information Engineering, Putian University, Putian 351100, China

**Keywords:** biomass, electromagnetic wave, hierarchical porous, synergistic loss mechanism

## Abstract

Hierarchical-porous-structured materials have been widely used in the field of electromagnetic wave (EMW) absorption, playing a critical role in minimizing EMW interference and pollution. High-quality EMW absorbers, characterized by a lower thickness, lighter weight, wider absorption band, and stronger absorption capacity, have been instrumental in reducing damage and preventing malfunctions in the automotive and aviation industries. The utilization of discarded nut shells through recycling can not only alleviate environmental problems but relieve resource constraints. Herein, a facile method for the preparation of hierarchical porous biomass carbon derived from abandoned Xanthoceras Sorbifolium Bunge Shell (XSS) biomass was developed for high-performance EMW absorption. The porous structures of XSS biochar were studied by using different levels of the K_2_CO_3_ activator and simple carbonization. The effect of K_2_CO_3_ on the EMW parameters, including the complex permittivity, complex permeability, polarization relaxation, and impedance matching, was analyzed. The best EMW absorption performance of the XSS biochar was observed at a mass ratio of activator-to-biomass of 2:1. A minimum reflection loss (RL_min_) of −38.9 dB was achieved at 9.12 GHz, and a maximum effective absorption bandwidth (EAB_max_) of up to 3.28 GHz (14.72~18.0 GHz) could be obtained at a 1.8 mm thickness. These results demonstrated that hierarchical porous XSS carbon was prepared successfully. Simultaneously, the prepared XSS biochar was confirmed as a potential and powerfully attractive EMW-absorbing material. The proposal also provided a simple strategy for the development of a green, low-cost, and sustainable biochar as a lightweight high-performance absorbing material.

## 1. Introduction

With the rapid development of electromagnetic wave (EMW) technology and its wide application in human communication, medical and defense security fields, etc., the problems caused by electromagnetic interference and pollution are becoming more and more serious [1,2,3]. EMW absorbers can prevent EMWs from interfering in the normal operation of electronic components. These absorbers can be widely used in smart phones, computers, microwave ovens, and other electronic products, providing a safer environment for people to use them in. It is worth mentioning that porous lightweight wave-absorbing carbon materials not only have outstanding application prospects in stealth and anti-radiation applications but also have a broad scope for development in other fields. For example, in aerospace, automotive manufacturing, and other fields, these materials can be used as fuel cells and electric vehicle conductive materials, improving energy efficiency and system performance; in the construction industry, porous lightweight wave-absorbing carbon materials can also be used for sound insulation and thermal insulation to enhance the quality of the indoor environment. Therefore, great efforts have been made to develop ideal absorbing materials with superior performance in terms of low thickness, light weight, wide absorption band, and strong absorption capacity [4,5]. The current methods for the enhancement in the EMW absorption performance are via compositing absorbing materials or designing hierarchical pore structures. Metal and/or magnetic materials are often induced into the composition of these absorbing materials [6,7,8]. Although this strategy has been successfully applied to achieve high impedance matching and EMW attenuation, it is limited in application due to its high cost, the higher density of materials, or the complex preparation process. To achieve easier and more economical EMW absorption, designing hierarchical pore structures is a good choice. First, hierarchical pores can change their structural hierarchy, volume, or pore size, providing a large surface area and rich pore structures that allow for a large degree of reflection and scattering of EMWs between the pore walls, increasing the path of incident EMWs and the probability of absorption and decay of the absorber [9]. Secondly, these hierarchical pore structures are widely available and relatively accessible. This approach is suitable for applications enhancing EMW absorption, but they often lack low-cost raw materials, simplicity, and green sustainability in terms of the preparation processes. Therefore, searching for alternative raw materials and developing a green, low-cost, and sustainable strategy for novel EMW materials is of great significance.

In recent years, utilizing abandoned biomass material has been attracting lots of attention in various fields because it can not only alleviate environmental problems but also relieve resource constraints. Biomass carbon can be derived from wood [10], leaves [11], seed shells [12], and others, whose natural porous structures, with their large pore volume and high surface area, can be preserved. Biomass carbon has been applied in the EMW absorption field due to its low density, high electrical conductivity, high flexibility, low cost, the fact that it is easy to obtain, and its excellent absorption ability [13]. While biomass carbon has natural three-dimensional pore structures, they lack a sufficient porous hierarchy for regulating the propagation paths of EMWs, solid–cavity interfaces for interface polarization, and defect centers for the formation of polarization sites [14,15]. As a result, the preparation of hierarchical porous biomass carbon would be an effective strategy for improving EMW adsorption. Therefore, it was hypothesized that controlling the microstructure of biomass carbon can be carried out through the use of a biomass precursor, carbonization, and/or an activator [16]. Zhou’s group compared the effects of KOH, NaOH, K_2_CO_3_, and ZnCl_2_ activators on the structure of pinecone-derived porous carbon [17]. The results demonstrated that the type of activator is vital to construct the shape and size of porous materials. By using the application of a potassium carbonate (K_2_CO_3_) solution as an activator, the RL_min_ of pinecone-biomass-derived carbon can be as high as −76.0 dB at a matched thickness of 2.1 mm, and the maximum EAB is 5.92 GHz at a matched thickness of 2.3 mm, covering a range from 11.92 to 17.84 GHz. Qiu and co-workers prepared different structures of porous biomass carbon derived from walnut shells by using KOH and different activation temperatures [18]. The best EMW absorption performance was achieved at an activation temperature of 600 °C, and the specific surface area was 736.2 m^2^/g. The RL_min_ at 8.88 GHz was −42.4 dB, and the EAB was only 1.76 GHz (8.08–9.84 GHz) with a matched thickness of 2 mm. Li and his co-workers used rice husk as raw materials, its RL_min_ reached −43.0 dB at 13.36 GHz, and the EAB was close to 3.68 GHz (11.44–15.12 GHz) at a thickness of 1.5 mm [19].

Despite the significant progress made in the above studies, the thickness and absorption properties of these materials still need to be further optimized. The above works show that the activator is significant for improving EMW absorption, but the sources of raw materials are also worth enriching. Xanthoceras Sorbifolium (XS) is widely distributed in the northern and northeastern areas of China. As a by-product of processing, hundreds of thousands of tons of Xanthoceras Sorbifolium Bunge Shells (XSSs) are produced, resulting in a large amount of biomass waste. In this work, waste XSSs as a model were used to prepare hierarchical pores, biomass carbon by activation, and simple carbonization processes. In order to prepare hierarchical pores and simplify the preparation process, anhydrous K_2_CO_3_ powder was used as an activator, and the amount of K_2_CO_3_ power was investigated. The relative complex permittivity (ε_r_ = ε′ − jε″), the relative complex permeability (µ_r_ = µ′ − jµ″), the reflection loss (RL) value, the thickness and effective absorption bandwidth (EAB), the attenuation constant (α), and the impedance matching (|Z_in_/Z_0_|) of XSS biomass carbon were all studied to assess EMW absorption performance. The results showed that the best EMW absorption of XSS biomass carbon was obtained by a mass ratio at 1:2. Under a filling ratio of 25 wt% XSS biomass carbon, the RL_min_ reached −38.9 dB and the EAB_max_ reached up to 3.28 GHz at a thickness of 1.8 mm. This work provides a new source of biomass that is low-cost and feasible for high-performance EMW absorption materials from waste biomass.

## 2. Results

### 2.1. Preparation of Hierarchical Pore XSS Biomass Carbon

For EMW absorption, the abundant pores and hierarchical pores will bring a large number of solid–gas interfaces, larger propagation paths of EMWs, and more defect centers, which can enhance interfacial polarization, multiple reflection, and scattering and increase polarization sites [20,21]. Thus, EMW absorption was enhanced. Traditional activators are generally KOH, NaOH, K_2_CO_3_, and ZnCl_2_ solutions for their alkaline etching, and the prepared pores are often mesopores. Using these traditional activators, the removal of water through the calcination process ultimately produces a large number of pores. Anhydrous K_2_CO_3_ powder was chosen as an activator for simplifying the process and preparing hierarchical pores in this work. According to the report [22], during the carbonization process, anhydrous K_2_CO_3_ was broken down into potassium oxide (K_2_O) and produced carbon dioxide (CO_2_) (Reaction (1)). Then, K_2_CO_3_ and K_2_O absorbed the carbon (C), following the formation of elemental potassium (K) and carbon monoxide (CO) (Reaction (2) and (3), allowing the successive conversion of C to CO (Reaction (4)). In addition, CO_2_ can also be absorbed on the solid porous carbon (C) to form CO (Reaction (4)). Therefore, it is deduced that micropores would be produced by the carbonization process, and mesopores are prepared for carbon oxidation. Finally, hierarchical porous XSS biomass carbon was prepared by the activator K_2_CO_3_ and pyrolysis.

K_2_CO_3_ = K_2_O + CO_2_, (1)

K_2_CO_3_ + 2C = 2K + 3CO, (2)

C + K_2_O = 2K + CO, (3)

CO_2_ + C = 2CO. (4)

### 2.2. Characterization of Hierarchical Pore

**Figure 1:**
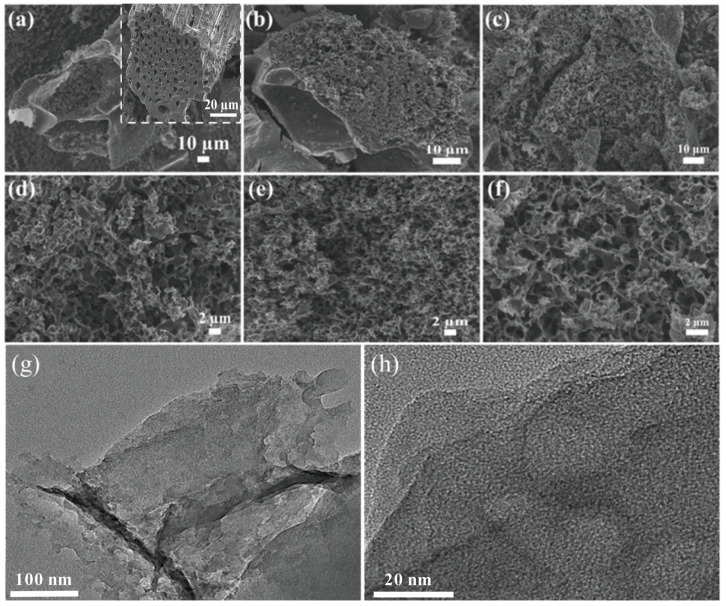
SEM images of different XSSs. (**a**,**d**) XSS-12, (**b**,**e**) XSS-22, (**c**,**f**) XSS-32, (**g**) TEM image, (**h**) HRTEM image.

Three types of XSS biomass carbon were prepared at a pyrolysis temperature of 700 °C, and FE-SEM, XRD, and Raman spectra were used to characterize the size and morphology of the material etched with different K_2_CO_3_ contents. The inset SEM image in Figure 1a shows that the unactivated XSS biomass carbon has tubular structures. Many irregular pores are distributed in the cross-section of tubes and their diameter is about 3–8 μm, which demonstrates that these structures can be fully preserved, although they are treated at high temperature. Furthermore, the multilayer stacked tubes and the pores among the tube walls are beneficial to the microwave absorption performance of the absorber. Relatively regular pores with ultrathin carbon sheets and interconnected circular-like pore structures in XSSs were found, as shown in Figure 1, indicating that it is successful in forming pores by using anhydrous K_2_CO_3_ as an activator, and inherited tube structures. Figure 1a,d also display the good hierarchical interconnectivity of pores in XSS-12. Compared with XSS-12, XSS-22, and XSS-32, it is easy to find the smallest pore size in XSS-12, and the densest in XSS-22, which further indicate that anhydrous K_2_CO_3_ is an effective activator and that too much K_2_CO_3_ would cause the collapse of small pores. Nevertheless, once the temperature is over 750 °C or at a slow heating rate, the XSS biomass carbon particles would become larger and the XSS biomass tube and irregular pore structures would be destroyed at the same time. In addition, the slow heating rate induced a high energy dissipation and long pyrolysis time. Moreover, when the pyrolysis temperature is below 700 °C or the heating rate is overly fast, the activator K_2_CO_3_ in the XSS biomass cannot be completely decomposed, generating insufficient pore structures and eventually resulting in poor EMW absorption efficiency. The TEM image shows that the unmodified material has abundant and fine pores. In addition, the HRTEM image shows that there are no lattice stripes in the unmodified XPC-700, which is an amorphous structure of activated carbon.

**Figure 2:**
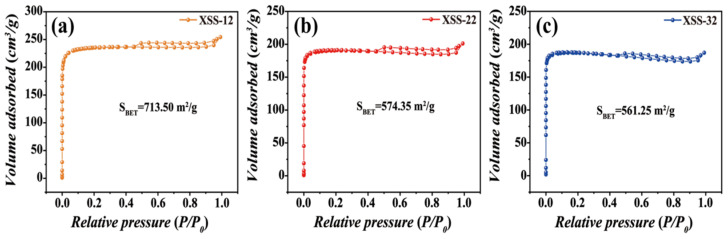
N_2_ adsorption–desorption isotherms of XSS. (**a**) XSS-12, (**b**) XSS-22, (**c**) XSS-32.

To further understand the prepared pore, the pore volume and pore size were analyzed by N_2_ adsorption–desorption isotherms in Figure 2. The typical IV curves with H_2_ hysteresis gyrus curves suggested that three types of XSS biomass carbon all have micropores and mesopores [23], further verifying that the hierarchical pores are successfully prepared in XSS biomass carbon. It is worth noting that the hysteresis loop of XSS-12 biomass carbon shifted towards a higher pressure than the other two, indicating that it has a larger pore size. The specific surface area, pore volume, and pore size distribution of XSS biomass carbon were calculated and are shown in Table 1. The largest specific surface areas, pore volume, and pore size are XSS-12, which are 713.5 m^2^/g, 0.39 cm^3^/g, and 2.06 m^2^/g, respectively. This suggests that the shrinkage and collapse of nanopores goes up with the increase in anhydrous K_2_CO_3_ during the carbonization process.

### 2.3. Characterization of XSS Biomass Carbon

**Figure 3:**
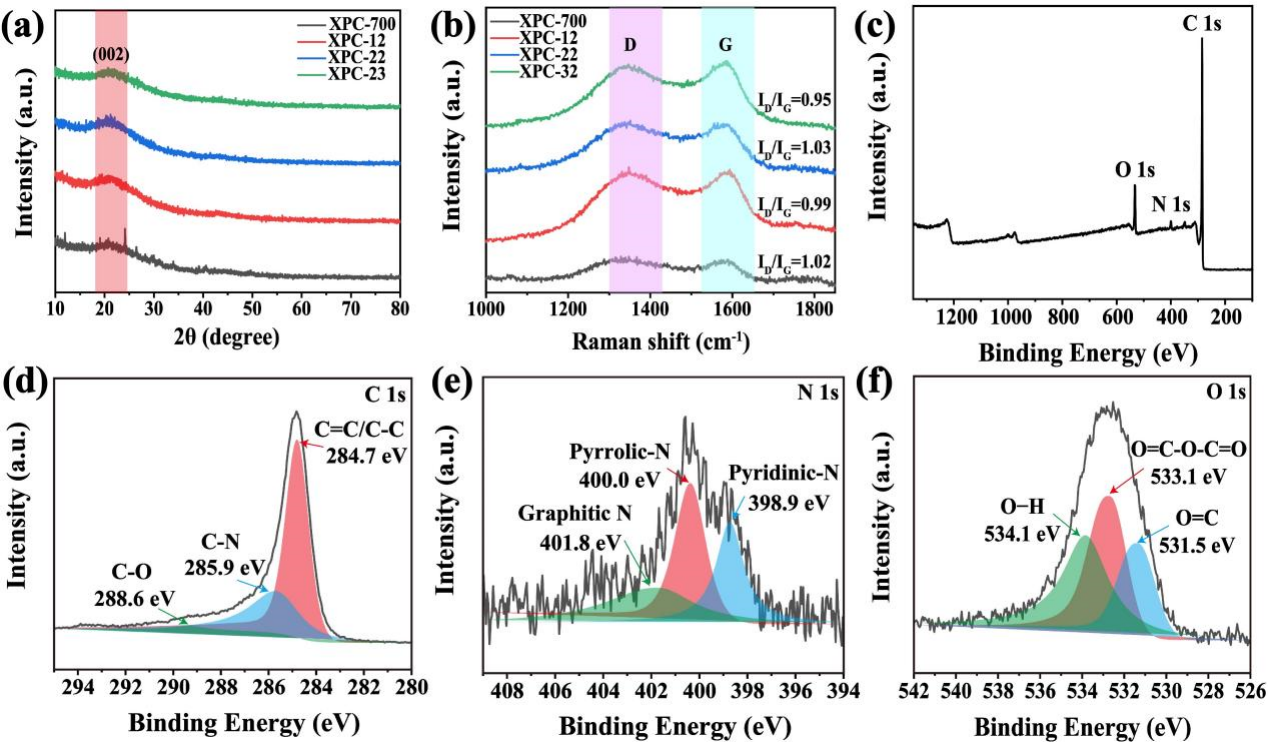
Characterization of XSS biomass carbon: (**a**) XRD patterns, (**b**) Raman spectra, (**c**) full spectrum of XPS, (**d**) C 1s, (**e**) N 1s, (**f**) O 1s. D is D-band and G is G-band.

To further investigate the effect of K_2_CO_3_ on crystal structures and state properties of XSS biomass carbon, wide-angle XRD and Raman spectra were used. In Figure 3a, all samples show broad diffraction peaks at 22.7°, corresponding to the (002) crystal plane of the graphitized carbon. The presence of broad diffraction peaks indicates that these samples have amorphous characteristics and are amorphous structural carbon [24,25]. In addition, with the rise in the K_2_CO_3_ content, the diffraction peak gradually increases, which indicates that the degree of amorphous XSS biomass carbon gradually goes up subsequently [26]. In Figure 3b, all samples have distinctive characteristic peaks near 1345 cm^−1^ (D-band) and 1575 cm^−1^ (G-band), demonstrating that the prepared biomass carbon contains disordered or defective sp^3^ hybridized carbon atoms (D-band) and disordered or defective sp^2^ hybridized carbon atoms (G-band) [27]. In addition, the I_D_/I_G_ ratios of XSS-12, XSS-22, and XSS-32 were 0.99, 1.03, and 1.05, respectively. In brief, the I_D_/I_G_ values varied with the increase in the activator K_2_CO_3_, indicating that activator K_2_CO_3_ has a significant impact on the graphitization degree of XSS biomass carbon. The reason is speculated that with the increase in K_2_CO_3_, the pore of XSS biomass carbon is contracted and collapsed; afterwards, the graphite crystal carbon is destroyed consequentially.

We further investigated the composition and oxidation states of the XSS biomass carbon by XPS. In Figure 3c–f, the XPS full spectrum indicates that C, N, and O elements are present in XSS biomass carbon. The C1s spectra can be divided into 284.7 eV, 285.9 eV, and 288.6 eV, which correspond to C=C/C-C, C-N, and C-O [28]. N1s were distributed with three peaks at 398.9, 400.0, and 401.8 eV, corresponding to pyridine nitrogen, pyrrole nitrogen, and graphite nitrogen. The XPS spectra of O 1s have three peaks at 531.5, 533.1, and 534.1 eV for C=O, O=C-O-C=O, and O-H, respectively [29]. The presence of C=O bonds indicated that oxygen-containing functional groups existed in the amorphous C, which would favor dipolar polarization.

### 2.4. EMW Absorption

The relative complex permittivity (ε_r_ = ε′ − jε″) and the relative complex permeability (µ_r_ = µ′ − jµ″) are two vital factors for EMW absorption performance. In general, the parameters of ε′ and µ′ represent the polarization and magnetization under electromagnetic field radiation [30]. The parameters of ε″ and μ″ demonstrate the loss and attenuation characteristics of the material in terms of conduction, dipole, resonance, or associated relaxation under electromagnetic field radiation [31]. In addition, the dielectric loss capability and magnetic loss capability can be evaluated by tan δe = ε″/ε′ and tan δm = µ″/µ′, respectively [32]. The ε′ and ε″ values are only in the appropriate range for the material to obtain absorbing behavior. The reasonable ranges of ε′ and ε″ values are 5~20 and 1~10, respectively. When the dielectric constant is too high (ε′ > 20, ε″ > 10), most of the incident waves will be reflected on the surface of the material. When the dielectric constant is too low (ε′ < 5, ε″ < 1), the incident waves will pass through the material completely [33]. If the sample has weak or no magnetism, µ′ and µ″ are around 1 and 0, respectively.

**Figure 4:**
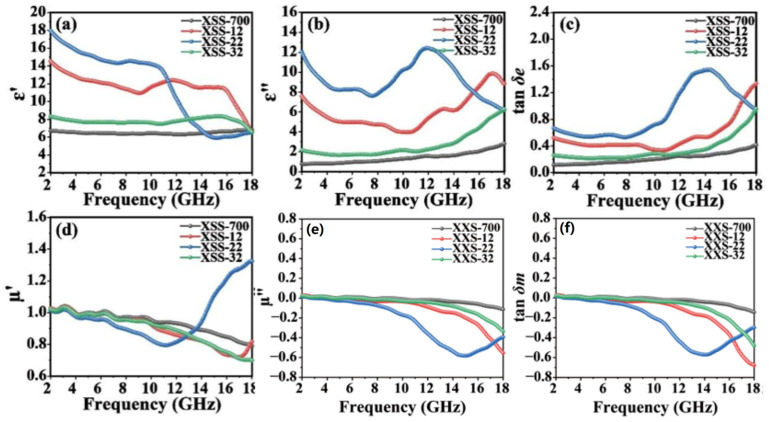
Electromagnetic parameters of XSS biomass carbon: (**a**) real part of complex permittivity, (**b**) imaginary part of complex permittivity, (**c**) tangent of dielectric loss, (**d**) real part of complex permeability, (**e**) imaginary part of complex permeability, (**f**) tangent of magnetic loss.

Figure 4 illustrates the effect of K_2_CO_3_ on the electromagnetic parameters of XSS biomass carbon. The ε′ values of XSS-700 and XSS-32 are basically constant with frequency in the range of 2~18 GHz, and the ε″ values remain constant and then increase slowly with frequency in Figure 4a–c. The ε′ hits its maximum value when the frequency of XSS-22 is situated in the range of 5 to 11. Nevertheless, the ε″ value of XSS-22 is greater than 10, which is not conducive to the EMW absorption. The ε′ and ε″ values of XSS-12 are both within reasonable ranges and better than other values. Combined with other characterizations, the smaller the content of activator K_2_CO_3_, the larger the S_BET_, and the greater the number of pores inside XSS-12 biomass carbon. According to Maxwell–Wagner polarization theory [34], the larger number of pores can generate more inhomogeneous solid–gas interfaces, resulting in an increasing interfacial polarization, which drives the aggregation of free charges under alternating electric fields, resulting in more polarized charges, which can increase the dielectric constant. Hence, XSS-12 has the best dielectric loss capacity. The trend of the dielectric loss tangent (tan δe) value is similar to the direction of the ε″ value, indicating that the complex dielectric constant ε″ is a major reason for the dielectric loss of XSS biomass carbon. The parameters related to the complex permeability and magnetic loss are shown in Figure 4d–f. No magnetic components were introduced in these three samples. So µ′ and μ″ should be around 1 and 0, respectively. For μ″, all three samples are negative at high frequencies, indicating that some magnetic energy radiates from the sample [35]. The magnetic loss of the three samples is much lower than the dielectric loss, indicating that the dielectric loss plays a dominant role in these materials.

**Figure 5:**
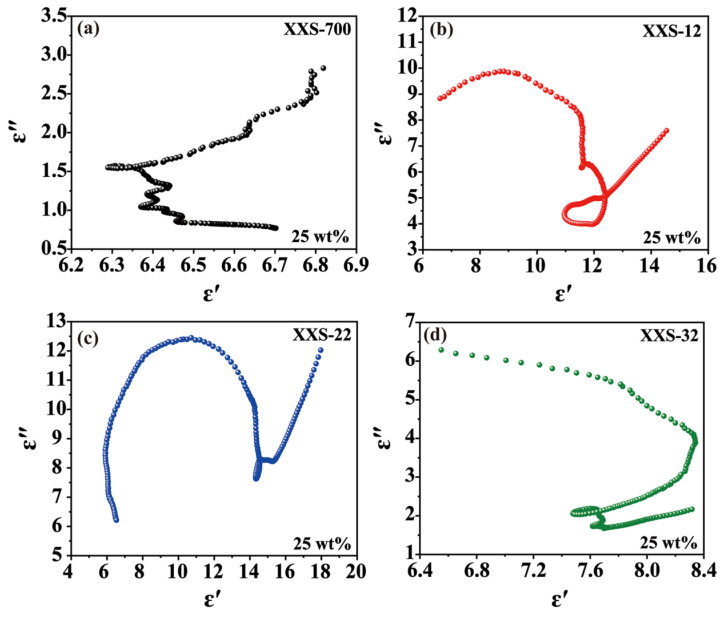
Relationship between ε′ and ε″ (Cole–Cole diagram) of porous carbon materials: (**a**) XSS-700, (**b**) XSS-12, (**c**) XSS-22, (**d**) XSS-32.

The dielectric loss is mainly caused by polarization and conduction loss. The Debye medium relaxation formula model (Cole–Cole model) can be used to describe the dielectric loss mechanism of these samples. More Cole–Cole semicircles indicate the strong polarization relaxation process in the decaying EMW process [36]. The long tail and each semicircle represent the conduction loss and the relaxation process [37]. As shown in Figure 5, XSS-12 has more and larger semicircles than the others, indicating that XSS-12 has stronger polarization loss and conduction loss. In addition, the tail of XSS-12 is larger than the semicircles, suggesting that conduction loss replaces polarization loss as the main loss mechanism. The larger dielectric loss was deduced from the larger specific surface areas resulting from the lower level of anhydrous K_2_CO_3_. In view of complex permittivity, complex permeability, and polarization relaxation, the mass ratio of K_2_CO_3_ to XSS of 1:2 has the best EMW absorption.

**Figure 6:**
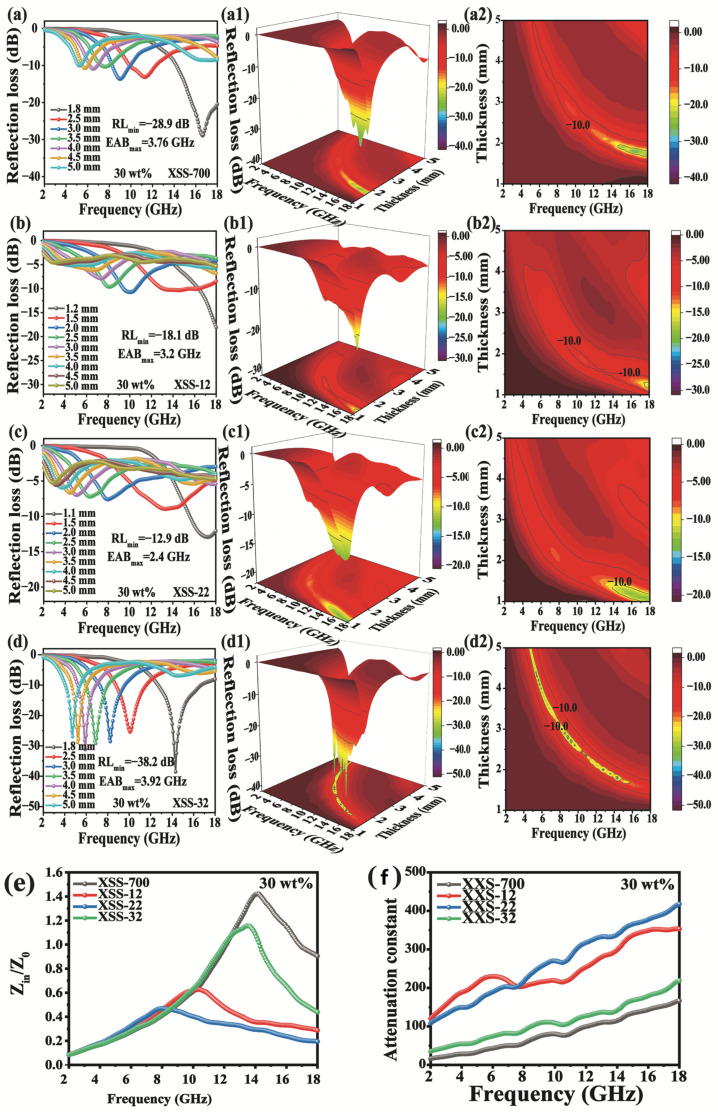
Reflection loss curve of XSS material: (**a**,**a1**,**a2**) XSS-700, (**b**,**b1**,**b2**) XSS-12, (**c**,**c1**,**c2**) XSS-22, (**d**,**d1**,**d2**) XSS-32, (**e**) attenuation constant diagram, and (**f**) impedance matching diagram of XSS biomass carbon with a thickness of 2.6 mm.

The RL value, thickness, and EAB are the three traditional golden criteria for examining EMW absorption performance. When RL is less than −10 dB, it has a 90% absorption efficiency of EMWs. As shown in Figure 6a–d, (a1–d1) and (a2–d2), the lowest RL_min_ is XSS-700, whose value is only −14.1 dB, indicating that XSS-700 has the worst absorbing performance, which can be attributed to the poorest dielectric loss. When K_2_CO_3_ was activated, the RL_min_ value was significantly increased. Since the activator causes hierarchical pores, the introduced defects and oxygen-containing functional groups lead to substantial interfacial polarization and dipolar polarization. All of them achieved the best performance of EWM absorption of XSS-12 with RL_min_ = −38.9 dB at 2.6 mm of thickness and an EAB of 3.28 GHz at 1.5 mm. In conjunction with the porous morphological characteristics of XSS biomass carbon, the effect of porosity on the EMW absorption performance can be easily found, indicating that the RL and EAB can be regulated by controlling the ratio of the activator to the biomass.

α and |Z_in_/Z_0_| are two key factors of EMW absorption materials, which can be used to further understand the improvement mechanisms of EWM absorption. α indicates the EMW attenuation ability of the absorbing material, and the larger α is, the stronger the EMW attenuation. |Z_in_/Z_0_| refers to the ability of incident EWM waves to enter absorbing materials. If the value of |Z_in_/Z_0_| is close to 1, that indicates a good absorption performance. As shown in Figure 6e, it is easily found that the performance of α is in the sequence of XSS-22 > XSS-12 > XSS-32 > XSS-700, while the |Z_in_/Z_0_| of XSS-22 is worst in Figure 6f. The |Z_in_/Z_0_| values of XSS-700, XSS-22, and XSS-32 deviated more from 1, indicating that poor impedance matching equally leads to poor absorption performance. The |Z_in_/Z_0_| value of XSS-12 is closer to 1 in the frequency range of 6–10 GHz, and XSS-12 has a strong absorption capacity for EMWs, which is favorable to dielectric loss, further indicating the good absorption performance of the sample.

In summary, the prepared XSS-12 with hierarchical pores has excellent EMW absorption performance due to dielectric loss as well as excellent |Z_in_/Z_0_|. Compared with other representative absorbers in Table 2, the prepared XSS-12 absorber has a significant RL, an ideal EAB, and a lower thickness. These results suggest that the biomass-derived porous carbon absorber is a lightweight and efficient absorber of EMWs, which furnishes an effective EMW absorber. Importantly, our proposal also provides an efficient strategy to recycle waste biomass, which not only resolves waste biomass and its environmental problems when being treated, such as harmful gases generated during waste biomass degradation, but also alleviates the pressure of an excess raw material supply. Hence, our work would provide a potential and valuable guideline for achieving a high-value-added utilization of biomass waste.

### 2.5. Mechanism of EMW Adsorption

**Scheme 1:**
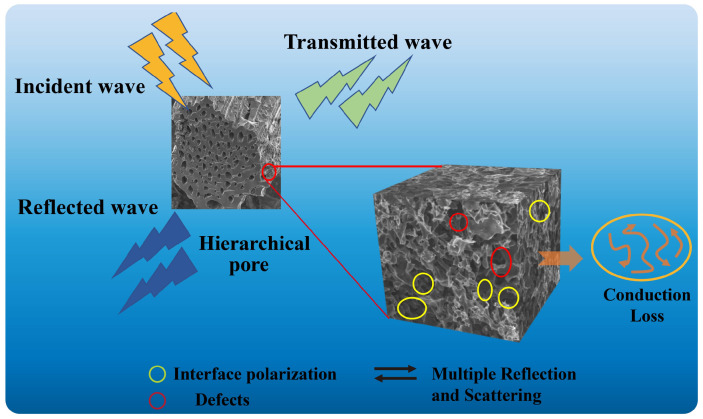
EMW absorption mechanism of hierarchical porous XSS biomass carbon.

The electromagnetic wave absorption properties of biomass carbon materials are closely related to their composition, surface chemical activity, microstructure, and pore size, which are mainly determined by biomass precursors and their carbonization or activation. As shown in Scheme 1, the main mechanisms are as follows: Firstly, the hierarchical pore in the XSS biomass carbon enables it to have good impedance matching characteristics, which enables more EMWs to enter the XSS absorbing material and cause multiple reflection and scattering. Secondly, hierarchical pores in the shell can be interconnected to construct a three-dimensional carbon skeleton, generate a conductive network, and enhance conductive loss and EMW attenuation. Finally, hierarchical pore biomass carbon is abundant in defects, which is conducive to dipole polarization. In addition, hierarchical pore biomass carbon provides abundant pores and heterogeneous interfaces, which make the porous carbon/air and porous carbon/paraffin interfaces accumulate a lot of charges, resulting in interface polarization. In a word, the porous structure not only provides the material with a larger specific surface area and inhomogeneous interfaces (air and interfaces), but it also enables multiple reflections, scattering, and interfacial polarization. These properties contribute to efficient energy dissipation, converting electromagnetic wave energy into heat or other forms of energy during prolonged propagation. The synergistic effect of these loss mechanisms improves the EMW absorption performance in XSS biomass carbon.

## 3. Materials and Methods

### 3.1. Reagents and Materials

XSSs came from Jingtai County, Baiyin, China; they were washed repeatedly with distilled water and dried in an oven at 80 °C; and then they were crushed into a powder to use. Anhydrous potassium carbonate (K_2_CO_3_) and hydrochloric acid (HCl, 37%) were purchased from Shanghai Maclean Biochemical Technology Co., Ltd. (Shanghai, China) All the chemicals were analytically pure and used without further purification.

### 3.2. Preparation of XSS Biomass Carbon and EMW Absorber

**Scheme 2:**
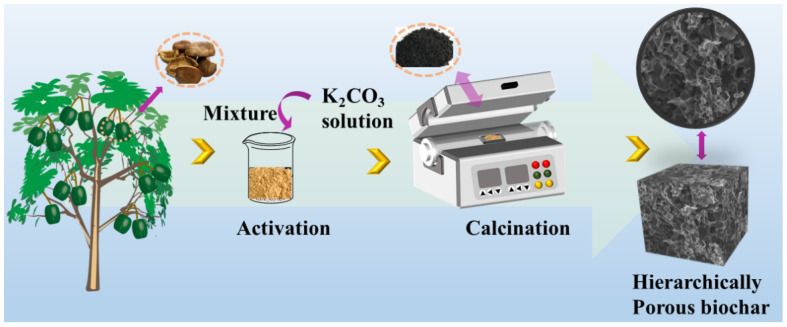
The schematic illustration of the synthesis of XSS-derived porous carbon.

The XSS biomass carbon was prepared in a fixed procedure according to the previous study [7]. We thus focused on the amount of activator. In brief, XSSs were washed three times with distilled water and dried at 80 °C, following crushing into a powder in Scheme 2. The prepared XSS powder was passed through 100 mesh sieves. Then, the mass ratios of anhydrous K_2_CO_3_ to the XSS powder were optimized at 1:2, 2:2, and 3:2, named XSS-12, XSS-22, and XSS-32. The XSSs without anhydrous K_2_CO_3_ were named XSS-700. The mixture was loaded in a quartz boat and calcined under N_2_ protection at 700 °C for 2 h with a heating rate of 2 °C/min. After cooling to room temperature, the products were washed with deionized water and the 1 mol/L HCl solution until the filtrate was neutral. The samples were dried at 60 °C and maintained at room temperature. Finally, the prepared XSS biomass carbon was homogeneously dispersed in a paraffin matrix with a fraction of 25 wt% and 30%, and then pressed into a cylindrical mold with φ_out_ of 7.0 mm and φ_in_ of 3.04 mm for the determination of EMW absorption.

### 3.3. Characterization and EMW Analysis

The morphology of the samples was characterized by a field emission scanning electron microscope (FE-SEM, Ultra55, Zeiss, Jena, German). The phase compositions of the samples were analyzed by using an X-ray power diffractometer (XRD, Rigaku, Smart Lab, Akishima, Japan). The state of carbon was measured by a Raman spectrometer (Renishow, In Via, Wotton-under-Edge, UK) at 532 nm of excitation wavelength. The surface chemical valence state of the sample was measured using X-ray photoelectron spectrometry (XPS, K-alpha, ThermoFsher Scientific, Tokyo, Japan) with monochromatic Al Kα (1486.68 eV) radiation. The special surface area (S_BET_) and pore size were determined by a nitrogen isothermal adsorption–desorption analyzer (Quantachrome, Autosorb-iQ3, Boynton Beach, FL, USA). The EWM parameters of samples were tested by a vector network analyzer (VAN, Agilent PNA N5222A, Santa Clara, CA, USA).

## 4. Conclusions

The XSS biomass carbon was prepared by a simple activation carbonization process to absorb EMWs. By adjusting the mass ratio of activator K_2_CO_3_, different hierarchical pores of XSS biomass carbon can be achieved. The hierarchical pores can effectively regulate the dielectric constant and impedance matching to improve EMW performance. These results show that with a filling rate of 25 wt%, XSS-12 has an RL_min_ of −38.9 dB and an EAB of 3.28 GHz (14.72–18.0 GHz) at a matched thickness of 2.6 mm and a frequency of 9.12 GHz. The effective utilization of the XSSs can not only relieve resource constraints but also greatly reduce the environmental pollution caused by the disposal of waste. The proposal provided a simple, green, low-cost, sustainable strategy for the development of high-performance absorbing material.

The carbon materials synthesized in our work show superior competitiveness for practical applications in electromagnetic wave absorption compared to porous carbon materials previously reported in the literature. Considering the problem of environmental pollution and treatment cost generated by renewable waste biomass globally, the present work provides an efficient and eco-friendly strategy to synthesize hierarchical porous carbon materials with excellent properties using waste Avena sativa shells as precursors. The excellent electromagnetic wave absorption properties provide a valuable guideline for achieving a high-value-added utilization of biomass waste.

## Figures and Tables

**Table 1:** Specific surface area, pore volume, and pore size distribution of XSS biomass carbon.

Sample	S_BET_ [m^2^/g]	Pore Volume [cm^3^/g]	Pore Size [nm]
XSS-12	713.50	0.39	2.20
XSS-22	574.35	0.31	2.17
XSS-32	561.25	0.29	2.06

**Table 2:** The EMW absorption properties of biomass-derived materials.

Sample	Filler Loading [wt%]	Thickness [mm]	RL_min_ [dB]	EAB [GHz]	Refs
Walnut shell	70	2.0	−42.4	2.2	[26]
Cotton	25	1.65	−51.2	4.4	[32]
Fe_3_O_4_/rGO	50	1.5	−30.0	4.0	[36]
Ni(OH)_2_/AC	50	6	−23.6	2	[38]
Fe_3_O_4_/PJBC	50	4	−39.5	5	[39]
Wheat straw	30	4.7	−43.6	3.3	[40]
Loofah sponge	30	2.7	−44.8	4.69	[41]
Spinach stem	30	2.7	−62.2	7.3	[42]
XSS-700	25	1.9	−14.1	1.52	This work
XSS-12	25	2.6	−38.9	3.28	This work
XSS-22	25	1.4	−19.7	2.32	This work
XSS-32	25	1.8	−37.9	2.8	This work
XSS-700	30	1.8	−28.9	3.76	This work
XSS-12	30	1.2	−18.1	3.2	This work
XSS-22	30	1.1	−12.9	2.4	This work
XSS-32	30	1.8	−38.2	3.92	This work

## Data Availability

Data are contained within the article.
